# Psychological Predictors of Precautionary Behaviors in Response to COVID-19: A Structural Model

**DOI:** 10.3389/fpsyg.2021.559289

**Published:** 2021-04-28

**Authors:** Martha Frías-Armenta, Nadia Saraí Corral-Frías, Victor Corral-Verdugo, Marc Yancy Lucas

**Affiliations:** ^1^Law Department, University of Sonora, Hermosillo, Mexico; ^2^Psychology Department, University of Sonora, Hermosillo, Mexico

**Keywords:** empathy, COVID-19, precautionary behaviors, health practices, anhedonia, impulsivity

## Abstract

The first lines of defense during an epidemic are behavioral interventions, including stay-at-home measures or precautionary health training, aimed at reducing contact and disease transmission. Examining the psychosocial variables that may lead to greater adoption of such precautionary behaviors is critical. The present study examines predictors of precautionary practices against coronavirus disease 2019 (COVID-19) in 709 Mexican participants from 24 states. The study was conducted via online questionnaire between the end of March and the beginning of April 2020, when the pandemic response was in its initial stages in Mexico. The instrument included demographic items, as well as scales assessing COVID-19-resembling symptoms, empathy, impulsivity, anhedonic depression, general health practices, alcohol consumption, and COVID-19-associated precautionary behaviors. Most participants reported adopting limited social distancing or other precautionary behaviors against COVID-19. The results of a structural equation model demonstrated that the presence of COVID-19 symptoms was related to impulsivity and general health behaviors. However, no direct association between precautionary behaviors and the presence of COVID-19 symptoms was found. In turn, precautionary behaviors were more prevalent among participants who reported higher empathy and general health behaviors and were inhibited indirectly by impulsivity via alcohol consumption. Furthermore, the model suggests that anhedonic depression symptoms have a negative indirect effect on precautionary behaviors via general health behaviors. Finally, impulsivity showed a negative direct effect on general health behavior. These results highlight the role that general physical health and mental health play on precautionary behavior and the critical importance of addressing issues such as depression, general health behaviors, and impulsivity in promoting safe actions and the protection of self and others.

## Introduction

The coronavirus disease 2019 (COVID-19) pandemic has highlighted the salience of individual behavioral response to external threats such as an acute infectious disease outbreak. Approximately 1 year following initial public efforts to reduce the spread of coronavirus, more than 2.4 million deaths and 100 million cases have been confirmed worldwide (Johns Hopkins University, 2021). The virus represents a serious threat in an increasingly interconnected global society where behaviors in one location can impact public health in others. However, illness behaviors, which can be understood as the actions employed by an individual when evidence of disease appears (Boltz et al., 2013; Wiebe et al., 2018), do not appear to manifest uniformly across locations and cultures (Huynh, 2020).

Examining the underpinnings of illness behaviors contributes to burgeoning research into relationships between psychological factors and health actions. Furthermore, a focus on such relationships during the nascent stages of a specific threat like COVID-19 can provide insight into individual action prior to coordinated, official public health response. This study took place prior to effective testing and tracking of coronavirus when people were expected to recognize symptoms and self-quarantine accordingly and focused on historically understudied populations outside the United States and Europe. Thus, it has the potential to identify cultural/contextual nuance and contribute to investigatory diversity. This exploratory study probes psychological (empathy, anhedonia, and impulsivity) and behavioral (general health behaviors and alcohol consumption) factors that may influence precautionary behaviors during the initial stages of a pandemic event in a sample of Mexican participants.

Psychological factors may be particularly relevant as research has demonstrated not only predictive utility but also potential for promoting such factors to elicit prosocial actions. Emotion represents a variable that may influence risk perception, which may in turn guide judgment and action. Strong, negative emotional reactions such as fear may lead people to ignore factual information about the pandemic or to focus more on information that challenges scientific or governmental positions on COVID-19 (Bavel et al., 2020). Empathy, on the other hand, has been identified as a predictor of precautionary behavior that can be induced to promote such actions (Sassenrath et al., 2016; Pfattheicher et al., 2020). Inversely, factors such as anhedonia and impulsivity may exacerbate the negative effects of, or be exacerbated by, stressful events like the pandemic (Gaygısız et al., 2017; Reinders Folmer et al., 2020b).

The pandemic overwhelmed health services across the globe; the official and unofficial efforts for reducing contagion focused on promoting physical social distancing, washing hands, and other behaviors such as avoiding touching surfaces and faces. At the time of data collection, it was estimated that nearly one-third of humanity was under “lockdown” (ranging from mandatory full quarantine to non-mandatory public health recommendations) with nine in 10 living in a country with some form of travel restriction (Pew Research Center, 2020). However, the number of cases has continued to rise worldwide, suggesting a lack of compliance with measures recommended or required by governments and international health organizations. It is crucial to investigate factors that relate to compliance with health measures aimed at preventing COVID-19 spread.

Evidence suggests variation of health-care behaviors across populations as well as individual and group responses to internal and external health threats. A study of health-care-seeking behaviors found that immigrants living near the border in the United States chose to return to Mexico for health treatment, even when insured in the United States, citing a distinctly “Mexican medical practice” and a desire to maintain their medical home base in a familiar context (Horton and Cole, 2011). Research suggests that such cultural determinants may also impact precautionary behaviors aimed at controlling infectious disease spread (Gaygısız et al., 2017). More recently, a cross-national study of social distancing found COVID-19 precautionary behaviors to be heterogeneous across countries (Huynh, 2020). Considering the wide range of illness behavior response, a one-size-fits-all approach promoting precautionary measures may not fully encompass the various factors that drive such behaviors.

This study makes a distinction between two types of illness behavior: general health behaviors and outbreak-specific precautionary behaviors. The former can be understood as habitual behaviors like diet and exercise, while the latter are behaviors specifically employed in response to an acute health threat such as sanitizing surfaces and hands, social distancing, and staying at home. Examined in concert, these two types of health-related behaviors provide a snapshot of how an individual cares for self and interacts with others. While these behaviors could be considered overlapping, they have been separated into distinct constructs to better understand relationships between habitual health actions and those specifically directed toward protecting against an acute threat.

Precautionary behaviors have demonstrated efficacy at containing the spread of COVID-19 (CDC, 2020) while individual general self-care behaviors like regular exercise and eating a healthy diet can help prevent, manage, or improve symptoms of minor illnesses without requiring direct medical attention, and in the case of an acute threat, adding burden to an already overwhelmed health infrastructure. General health behaviors also represent an important component of mental and physical health maintenance, potentially mitigating feelings of isolation associated with adherence to social distancing and stay-at-home recommendations (CDC, 2020). Studies suggest that such actions may improve quality of life and the ability to function in those suffering chronic disease (Baumann and Dang, 2012). Perhaps most relevant to mitigation efforts is that both types of behavior can be promoted and fostered in the context of a contagious disease outbreak. A study of older adults in Mexico following the 2008 H1N1 outbreak found that an intervention focused on self-care improved both general knowledge and precautionary behaviors regarding respiratory illness and transmission (Márquez-Serrano et al., 2012). Similarly, a COVID-19 study from Italy found that self-care behaviors were associated with general health (De Maria et al., 2020).

Extant evidence has demonstrated relationships between various psychological factors and health and disease (Wiebe et al., 2018). These types of psychosocial–behavioral interactions are particularly salient when examining individual choice to enact precautionary or general health measures. Psychological factors such as empathy, anhedonia, and impulsivity have previously demonstrated relationships with precautionary and general health behaviors (Hodges and Myers, 2007; Kessing et al., 2014; King et al., 2016; Bacon and Corr, 2020; Pfattheicher et al., 2020). Similarly, general health behaviors and alcohol consumption have been associated with both long-term and acute health behaviors (WHO, 2018; Arora and Grey, 2020). Limited research has focused on the degree to which individuals in Mexico adjusted their daily lives during the early stages of the COVID-19 pandemic and how those behaviors may relate to underlying psychosocial traits.

Empathy has been identified not only as a promising psychological factor for predicting precautionary behaviors but also as one that can be promoted or induced to increase frequency and/or effectiveness of such actions. Empathy is typically defined as the individual’s response to perceptions of the current experience of another or others (Hodges and Myers, 2007) and has previously demonstrated positive relationships with precautionary health behaviors during pandemic events. An investigation of H1N1 in India found an association between greater empathy and increased health precautions and vaccination (King et al., 2016). A study of health-care workers in Germany found affective empathy to have a causal relationship with hand hygiene behaviors and that inducing empathy increased hand sanitizer usage (Sassenrath et al., 2016). Similarly, a study conducted in the early stages of COVID-19 (before many precautionary measures were widely implemented) demonstrated that empathy was a basic motivator for social distancing in participants in the United Kingdom, the United States, and Germany. Empathy for vulnerable populations was specifically identified as a variable encouraging physical distancing. The study likewise reported that experimentally induced empathy was found to promote motivation to adhere to physical distancing (Pfattheicher et al., 2020). Conversely, psychological entitlement, a trait characterized by sentiments that the self is more deserving than others, was found to be predictive of non-compliance with health measures (Zitek and Schlund, 2021). Given these antecedent studies, we would expect individuals with greater self-report empathy to likewise report greater incidence of precautionary and general health behaviors.

Furthermore, reports have linked different psychological traits to differences in compliance with COVID-19 health measures (Bacon and Corr, 2020; Nofal et al., 2020). Impulsivity, which has been linked to an inability to constrain inappropriate behavior (Malesza and Ostaszewski, 2016) and to foresee the consequences of one’s actions (Crysel et al., 2013), is potentially relevant. A Turkish study performed during an outbreak of H1N1 demonstrated a relationship between impulsivity and precautionary behaviors (Gaygısız et al., 2012). More recently, research from the Netherlands found that impulse control influenced sustained compliance with COVID-19 mitigation measures (Reinders Folmer et al., 2020a, Reinders Folmer et al., 2020c). A study from the United States found that compliance depended upon self-control in conjunction with capacity and opportunity for rule breaking (van Rooij et al., 2020), while another found self-control to be directly associated with adherence to social distancing measures, particularly among individuals who perceived such adherence as difficult (Bieleke et al., 2020). Individuals characterized by Dark Triad traits (psychopathy, Machiavellianism, and narcissism), or antisocial behaviors that have been associated with impulsivity, were less likely to engage in preventative behaviors (Nowak et al., 2020; Zajenkowski et al., 2020; Miguel et al., 2021).

Anhedonia is another psychological variable that may have an influence on precautionary and self-care health behaviors. Although not widely studied in the context of infectious disease, anhedonia has been prospectively associated with poor self-care (Kessing et al., 2014). Positive affect has been linked to improved self-care in cardiac patients even while controlling for demographic and other clinical factors (Kessing et al., 2014). Inversely, deficiencies in pleasure may be important affective mechanisms underlying self-care behaviors such as physical activity (Leventhal, 2012). Diagnostically, anhedonia has been found to be the best psychosocial predictor of major clinical events (Denollet et al., 2008). More recently, an electronic health record network cohort study showed that patients with a history of psychiatric illness were at a higher risk of being diagnosed with COVID-19 (Taquet et al., 2021). As such, psychiatric symptoms, such as anhedonic depression, may represent a promising avenue for examining the relationship between mental health and trait and state health behaviors.

Alcohol consumption is another potential variable of focus given its association with health issues (Griswold et al., 2018) and potential for increased use in the context of lockdown and quarantine. A Polish study eliciting responses in the initial stages of the COVID-19 outbreak (March, 2020) found that participants who increased their consumption of alcohol following physical distancing measures reported greater difficulty coping with everyday activities, suffered greater rates of depression, and were less likely to adopt coping strategies such as positive reframing (Chodkiewicz et al., 2020). More generally, alcohol use has been linked to negative outcomes not only through its direct effects on health but also indirectly through its relationship with decreased treatment adherence and self-care (WHO, 2018). Increased alcohol use has been associated with decreased adherence to outpatient medication (Grodensky et al., 2012) as well as decreased self-care behaviors in diabetes (Ahmed et al., 2006) and hypertensive patients (Rittmueller et al., 2015). Psychological factors such as impulsivity have also been linked to alcohol consumption (Dick et al., 2010; Gray and MacKillop, 2014). Recent studies have linked increased alcohol consumption with impulsivity (Kreek et al., 2005; Clay et al., 2020) and thus may be a link between impulsivity and health behaviors.

Comprehensive examinations of the individual psychosocial factors that influence general health behaviors and behaviors related to acute disease threats like COVID-19 have not been widely undertaken especially in Latin America. Furthermore, a better understanding of the underlying psychosocial predictors of pandemic behavior as it relates to factors such as empathy, impulsivity, and anhedonia can elucidate how behaviors manifest themselves under acute threat. As such, this article attempts to develop an examination of the interplay between general health behaviors (such as general hygiene, regular exercise, and eating a healthy diet) and threat-specific behaviors (such as social distancing, sanitizing, and handwashing) in the face of a contagious disease outbreak (COVID-19).

Given the importance of understanding the relationship between physical and mental health and propensity to adopt precautionary behaviors that protect oneself and others, the main objective of the current study was to investigate the effect of psychological factors (empathy, impulsivity, and anhedonic depression) on precautionary and general health behaviors. Furthermore, the study examines whether alcohol consumption has a relationship with precautionary factors. Considering antecedent studies, we predict that individuals who score higher in reported rates of alcohol consumption, impulsivity, and anhedonia and lower in reported rates of empathy will likewise report reduced frequency of general health and pandemic-specific precautionary behaviors. The design of this study casts a wide net to probe some of the underlying psychological factors and social behaviors associated with precautionary response. Furthermore, this initial research was intended to identify potential future avenues of research into the psychosocial nuances of infectious disease response.

## Materials and Methods

### Participants

The data were collected using a snowball recruitment procedure. Study invitations were sent via email, text, and social media in keeping with physical distancing guidelines. Prior to participation, all participants were informed of the study aims, benefits, and risks before signing a digital consent form. The sample included 709 individuals from 24 of the 32 Mexican states. All participants at or above the legal age of consent (18) were eligible. Mean age was 35.5 (*sd* = 14.8), ranging from 18 to 81. Most of the participants self-identified as female (517), with 178 identified as male, and seven as non-binary, and seven preferred not to answer. Approximately one-third of the participants self-identified as married (33.2%), and more than half of the sample reported being single (58.8% single); the remaining reported being either divorced, widowed, or living in cohabitation (5.2, 1.5, and 17.7%, respectively). About one-third of participants reported high school (27.3%), college degree (35.4%), or a postgraduate degree (31.1%) completion, while less than 2% reported completing elementary or middle school as their highest level of completed education (0.3 and 1.6%, respectively). Reported income ranged from low to high relative levels (6.1% low income, 18.1% low to medium, 30.2% medium, 22.9% medium high, and 17.7% high). Less than half reported having a steady salaried income (44.9%).

### Procedure

The questionnaire was distributed between the end of March and the beginning of April, when the pandemic response was in its initial stages in Mexico. During this period, health and government officials had issued a “stay at home” request (#quedateencasa), but the recommendation was not mandatory. Likewise, health and government officials disseminated informational campaigns about COVID-19 and hygienic measures to avoid contracting and spreading the virus.

Groups from various Mexican academic institutions were contacted electronically and invited to participate in the study. Academic liaisons were asked to subsequently distribute the invitation to their networks. Data were collected using Qualtrics software. Approximately 3% of those who received the link declined to participate. All the procedures used in this study comply with the ethical standards of national and international human ethics committees and were approved by the University of Sonora Ethics Committee.

### Translation

The scale assessing empathy was translated to Spanish from the original English. After translation, the items were backtranslated to check for equivalence of meaning between source and target texts. Spanish-speaking researchers evaluated the Spanish-translated instruments prior to the start of the study to assess and improve reliability and validity.

### Instruments

The instruments used in this study were selected to assess a wide range of psychosocial variables. Socioeconomic factors (age, gender, alcohol use, education, and occupation) were assessed alongside psychological factors such as empathy, anhedonic depression (anhedonia), and impulsivity. Behavioral variables related to general health practices (such as diet and exercise) and pandemic-specific precautionary behaviors (handwashing, social distancing, etc.) were assessed as well as self-report of COVID-resembling symptomology at time of the questionnaire.

#### Sociodemographic Variables

Participants were asked to report their age, gender, monthly family income, marital status, highest level of completed education, and whether they received a steady salary. They were additionally asked about their religious practices and political orientation as well as their tobacco use.

#### Alcohol Consumption

Alcohol consumption was assessed using one Likert-style item. Participants were asked to report how many beverages they consume, on average, per occasion (1 = one to two beverages; 2 = three to four beverages; 3 = five or more beverages).

#### Empathy

Empathy was assessed using four items from the Loewen et al. (2009) Empathy Quotient, which, in turn, is a short form of Wakabayashi et al. (2006). Only the reversed scaled items were included, reported using a Likert-type scale (0–4). Items included “I find it hard to know what to do in a social situation” and “I often find it hard to judge if something is rude or polite.” Our in-house translation demonstrated acceptable reliability (α = 0.64).

#### Impulsivity

Impulsivity was assessed using eight items from the Corr and Cooper (2016) Reinforcement Sensitivity Theory Personality questionnaire. The instrument response scale ranged from 1 (it does not apply to me) to 5 (it absolutely applies to me). Items included “I always buy things impulsively” and “I recognize that I do thing without thinking.” This scale was previously translated to Spanish and validated (Espinoza-Romero et al., 2019) in Mexico, demonstrating acceptable internal consistency in both a student sample (α = 0.78) as well as our sample (α = 0.74).

#### Anhedonic Depression

Anhedonic depression was assessed using eight items from the from the Mini Mood and Anxiety Symptom Questionnaire (Mini-MASQ) scale (Casillas and Clark, 2000) (two positively keyed items and six reverse-keyed items). Participants responded to items like “I feel happy” and “I feel that I have a lot of things to do” using a 5-point Likert-type scale (1 = nothing to 5 = extremely). The scale has been previously validated in Mexico (Corral-Frías et al., 2019) and reported acceptable internal consistency and reliability (α = 0.83) consistent with the one reported here (α = 0.84).

#### General Health Practices

The general health practices scale included five items from a self-care instrument (Corral Verdugo et al., 2021) and two items addressing general health. The instrument used a 5-point Likert-type scale ranging from “never” (1) to “always” (5). The scale demonstrated acceptable internal consistency in our sample (α = 0.63).

#### Precautionary Behaviors

The precautionary behavior scale was specifically developed for this study to elicit responses on actions that protect oneself and others against infection and transmission of contagious diseases. It included six items assessing preventative behaviors that participants had engaged in during the previous 3 days. The first three Likert-type items assessed the number of times participants left their house in the previous 3 days as well as asking them to report on their social distancing behaviors and face-touching frequency while outside of the home. The fourth item assessed greeting techniques wherein participants reported how they greeted others outside of the home. Greetings that adhered to social distancing recommendations (greeted verbally or non-verbally from far) were awarded more points than riskier actions such as handshaking, hugging, and cheek kissing.

The remaining two questions were open-ended aimed at eliciting responses on safe home entrance and handwashing behaviors. Participants were asked to describe their behaviors upon returning to the home following an outing and to explicitly describe their handwashing behaviors. “The “safe home entrance” variable was quantified after content analysis and was the summation of up to nine different protective behavior categories (e.g., washing hands, taking off shoes, and using disinfectant). Likewise, the “handwashing” variable assessed whether participants self-reported taking sufficient time and used the appropriate handwashing techniques. Both variables were quantified using a codification procedure developed via content analysis procedures. Descriptions were tallied such that if participants self-reported taking enough time (e.g., two rounds of the “happy birthday” song, at least 20 s) and described using an appropriate technique (e.g., washing between fingers, thumbs, and top of hands). All responses were evaluated, and relevant categories were developed until saturation was reached (Saunders et al., 2018). The final two questions were qualitative in nature to best assess precautionary health knowledge reported by the participants in the initial stages of the COVID-19 response. This was not only to probe responses on behaviors based on health recommendations but also to potentially identify additional (safe or unsafe) behaviors thought to protect against the virus.

#### Coronavirus Disease 2019-Resembling Symptomology

A seven-item scale was used to self-report COVID-resembling symptoms. Participants detailed the extent to which they had experienced seven symptoms of the virus during the past week, using a Likert-type scale “none” (1) to “extreme” (5). Respondents were asked to report on the frequency of fevers of 38°C (100.4°F) or more, headache, dry cough, loss of smell, loss of taste, stomachache, and diarrhea within the previous 7 days.

### Data Analysis

Internal consistency reliability [Cronbach alpha and average inter-item correlation (AIC)] and univariate (means and standard deviations) analyses were performed using SPSS v.25. Likewise, frequency analyses were performed on categorical variables. Given that three scales were created for this study or were modified from the original, confirmatory factor analyses were performed to test the unidimensional nature of the scales (see Supplementary Materials).

Finally, a structural equation model analyzing the direct and indirect influences of psychological factors on COVID-19-related precautionary behaviors and resembling symptoms was specified and tested using the maximum likelihood robust estimation method using EQS.

In accordance with recommendations from Hau and Marsh (2004), we used parcels that were calculated by averaging items randomly within each construct, except in the case of empathy where parcels were created by subscales. The maximum likelihood robust method was used because although we have a large sample, a previously specified model, and independent observations, we did not meet the normal distribution of the data (Mardia = 67.95). This methodology and the residual-based tests are thought to be the most accurate methods for analyzing non-normal data for structural equation models (Bentler, 2007).

To evaluate if the data support the proposed hypothetical model, two types of fit index indicators (Bentler, 2007) were considered: practical and statistical. The statistical indicator used was Satorra–Bentler chi squared (χ^2^), which measures the difference between the proposed models and the saturated χ^2^. To make the χ^2^ test less dependent on sample size, we used the relative χ^2^, which is calculated by dividing the χ^2^ fit index by the degrees of freedom. Congruent with literature (Schumacker and Lomax, 2004), if this ratio is <5, we deemed the model to have good fit. Since statistical indicators are very sensitive to sample size, the following practical indicators were also considered: comparative fit index (CFI), Bentler–Bonnet non-normed fit index (BBNNFI), and root mean square error of approximation (RMSEA).

The theoretical model suggests that general health behaviors have a direct association with COVID-19-related symptoms. The model is based on previous literature, which found that general health behaviors (e.g., self-care) may help maintain physical and mental health and can, therefore, prevent COVID-19 symptoms (Baumann and Dang, 2012; Wiebe et al., 2018; De Maria et al., 2020). On the other hand, our model suggests that impulsivity will have a direct association with general health behaviors, given the logical causal order establishing that traits (impulsivity) affect behavior (general health behavior) (Hofmann et al., 2008). This is consistent with previous empirical reports establishing an association between impulsivity and general health behaviors. A Turkish study demonstrated this relationship in a study during the H1N1 pandemic in which recommended behaviors were predicted by impulsive sensation seeking (Gaygısız et al., 2012). However, given the cross-sectional design, the model cannot establish a causal relationship between psychological factors and health behavior.

**FIGURE 1:**
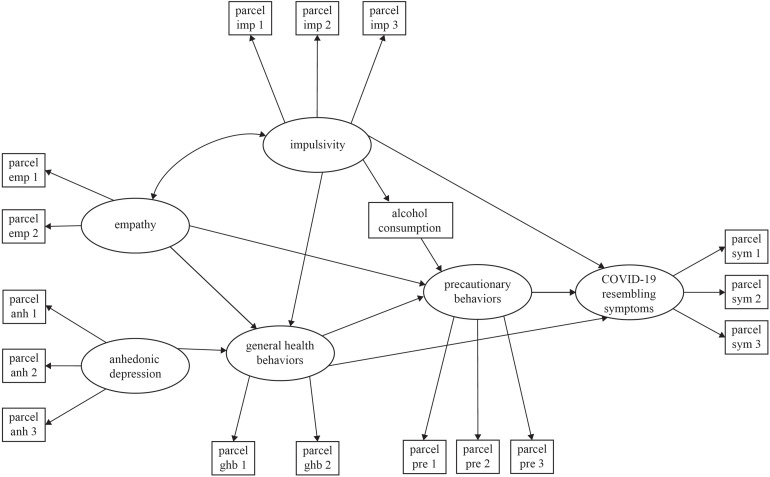
Theoretical model of predictors of precautionary behaviors.

Our model proposes that “impulsivity” and “anhedonia” will have an indirect effect on “precautionary behaviors” and “COVID-19-resembling symptoms” via “general health behaviors.” Furthermore, “empathy” will have a direct effect on “precautionary behaviors.” This model is based on previous evidence demonstrating that impulsivity and anhedonia influence trait health behaviors, and these may lead to better health-related responses (state) in the face of a health crisis such as COVID-19. In Figure 1, we present a hypothetical model based on the previously presented literature. The model predicts that individuals who report higher impulsivity (Reinders Folmer et al., 2020a,c; van Rooij et al., 2020) and anhedonia (Denollet et al., 2008; Taquet et al., 2021) and report lower rates of empathy (Pfattheicher et al., 2020) will report reduced frequency of general health and pandemic-specific precautionary behaviors. We also hypothesize that impulsivity will have an indirect effect on pandemic-specific precautionary behaviors through alcohol consumption (Clay and Parker, 2020; Kreek et al., 2005). Finally, we predict that general health and pandemic-specific precautionary behavior, as well as impulsivity, will have positive and direct effects on COVID-19-resembling symptoms.

## Results

The most reported COVID-resembling symptoms were headaches (43.1%), followed by stomachaches (26.6%) and dry coughs (17.6%) (see Supplementary Table 1). Within our sample, only 26.3% of participants avoided going out in the three previous days before completing the questionnaire, while 34.3% went out between two and five times (see Supplementary Table 2). The most reported reasons for going out were to buy food (52.3%), to work (18%), to visit relatives (12.3%), and to acquire medicines (10.2%) (see Supplementary Table 3). Seventy-eight percent of respondents admitted they consume alcohol, to different degrees, with 36% reported drinking three or more alcoholic beverages each time.

Table 1 shows the internal consistency and univariate statistics (means and standard deviations) for each of the instruments. The scales showed acceptable internal consistency reliability (α = 0.60–0.84) for most scales. The exception was the COVID-resembling symptoms measure (α = 0.57), which is not surprising given the range of symptoms associated with COVID-19. Since the Precautionary Behaviors measure included items with diverse codification (ranges of response: 1–5, 1–4, –3 to 2, 0–7, and 0–6), we used AIC to estimate reliability. The scale produced an AIC = 0.16, which is considered acceptable (Briggs and Cheek, 1986; Clark and Watson, 1995).

**TABLE 1:** Reliability and univariate statistics of scales (scale range of responses: 1–5).

**Scale/items**	**Mean**	***SD***	**Alpha**
General health practices			0.60
Does physical activity regularly to maintain health.	3.17	1.07	
Tries to consume healthy food.	3.75	0.76	
Visits doctor if feeling sick.	3.64	1.01	
Engages in practices of personal hygiene.	4.74	0.51	
Rests to recover health and energy.	4.24	0.78	
Generally, his/her health is good.	3.43	0.86	
Impulsivity			0.74
I think I should “stop and think” more instead of jumping into things too quickly.	3.01	0.99	
I sometimes cannot stop myself talking when I know I should keep my mouth closed.	2.22	1.01	
I often do risky things without thinking of the consequence.	1.89	0.91	
I find myself doing things on the spur of the moment.	2.16	0.94	
I’m always buying things on impulse.	2.01	0.99	
I would go on a holiday at the last minute.	2.14	1.13	
I think the best nights out are unplanned.	2.89	1.15	
If I see something I want, I act straight away.	2.22	0.96	
Empathy			0.64
I find it hard to know what to do in a social situation.^+^	1.41	0.99	
I often find it hard to judge if someone is rude or polite.^+^	1.07	1.00	
It is hard for me to see why some things upset people so much.^+^	2.10	0.91	
Other people often say that I am insensitive, though I don’t always see why.^+^	1.80	0.95	
Anhedonic depression			0.83
Felt really happy.^+^	2.94	0.98	
Felt like I was having a lot of fun.^+^	3.40	1.05	
Felt like I had a lot of energy.^+^	2.76	1.07	
Felt really lively, “up,”^+^	3.75	0.97	
Felt like I had a lot of interesting things to do.^+^	3.05	1.16	
Felt like I had a lot to look forward to.^+^	3.34	1.05	
Felt withdrawn from other people.	2.77	1.28	
Felt like nothing was enjoyable.	2.03	1.05	
COVID-19-resembling symptoms			0.57
Fever	1.02	0.21	
Headache	1.66	0.86	
Dry cough	1.25	0.58	
Sense of smell loss	1.10	0.40	
Sense of taste loss	1.06	0.30	
Stomach ache	1.37	0.72	
Diarrhea	1.20	0.58	

*COVID-19, coronavirus disease 2019. ^+^Reverse-keyed items.*

Participants reported limited implementation of social distancing and other precautionary measures against COVID-19. Most participants stood closer than 2 m from other people (78.8%), and almost all acknowledged touching their faces while outside their home (90.8%). The self-reported average social distance while out was between 1 and 1.5 m, and participants acknowledged touching their faces between three and five times in average. Most people reported hygienic greeting practices such as verbal and gestural greeting (*n* = 438), but also a few reported giving handshakes, kissing, and hugging (*n* = 68), while 114 did not find any acquaintances to greet while out. Participant took an average of 1.89 safe steps to enter their home after being out (range 0–7; where the most common was handwashing, *n* = 443). Likewise, participants described using an average of 1.98 different techniques (most mentioned thorough washing technique *n* = 308 and the use of soap *n* = 391) for effective handwashing (see Table 2).

**TABLE 2:** Reliability and univariate statistics of precautionary behavior scale.

**Items**	**Mean**	***SD***	**Min**	**Max**	**Alpha/AIC**
					0.54/0.16
Times went out home	3.26	0.92	1	5	
Social distancing	2.07	0.75	1	4	
Times touched face	3.18	0.91	1	4	
Hygienically greeted	0.72	0.83	−3	2	
Steps that followed at entering home	1.89	1.22	0	7	
Hands washing procedure	1.98	1.19	0	6	

*AIC, average inter-item correlation*.

**FIGURE 2:**
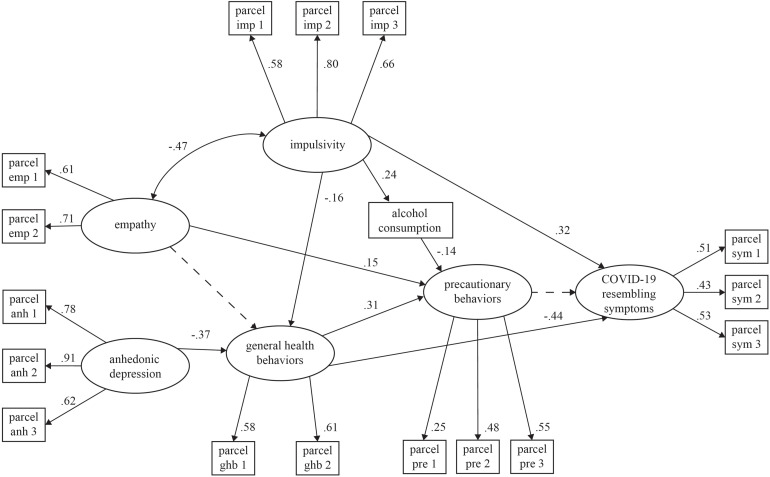
Structural model of predictors of precautionary behaviors. All factor loadings, structural coefficients and covariances are significant (*p* < 0.05), excepting the one marked with the dotted line. Goodness of fit: Satorra–Bentler χ^2^ = 217.41 (108 df), *p* < 0.001; relative χ^2^ = 2.01; BNNFI = 0.92, CFI = 0.94; RMSEA = 0.03.

Figure 2 shows the results from the structural equation model. All parcels loaded significantly (*p* < 0.05) on their factors, suggesting convergent construct validity for the used measures. Impulsivity directly negatively influenced health practices (β = −0.16) as well as COVID-19-resembling symptoms (β = 0.32) and indirectly influenced precautionary behaviors through alcohol consumption (β = 0.24), where alcohol had a negative effect on these behaviors (β = −0.14). Furthermore, it had negative covariance with empathy (β = −0.47). Anhedonic depression directly negatively impacted general health practices (β = −0.37). Precautionary behaviors were directly predicted by general health practices (β = 0.31) and empathy (β = 0.15). Finally, COVID-19-resembling symptoms were also directly and negatively impacted by general health practices (β = −0.44). The model showed acceptable goodness of fit (Satorra–Bentler χ^2^ = 217.47 [108 *df*], *p* < 0.001; relative χ^2^ = 2.01, *BBNNFI* = 0.92, *CFI* = 0.94, *RMSEA* = 0.03). This model explained 23% of the total variance of general health behaviors, 26% of self-reported COVID-19-resembling symptoms, and 18% of precautionary behaviors (see Figure 2).

## Discussion

This study examined psychological factors associated with precautionary COVID-19-related practices in a Mexican sample during the initial stages of pandemic response. Our data show that despite the “stay-at-home” recommendation, only few participants complied with the stay-at-home guidelines (26%) at the beginning of the pandemic. Although “work” was one of the main reasons for going out, respondents also mentioned buying food, visiting relatives and friends, acquiring medicine, and exercising outdoors. These rates are consistent with Google (2020)-generated reports of only a 27% reduction in mobility to workplaces in Mexico from March 15 (the day the national emergency was issued) to April 05.

As our hypothetical model proposed, in congruence with previous literature (Reinders Folmer et al., 2020a,c; van Rooij et al., 2020), our results showed that COVID-resembling symptoms were directly associated with impulsivity and general health behaviors, suggesting that people who are less impulsive and take regular care of their health experienced fewer COVID-19-resembling symptoms. We expected a direct effect of precautionary measures on COVID-19-resembling symptoms; however, we did not find a significant link. This may due to the relatively nascent stages of the viral spread in Mexico when data were collected.

The present study makes a distinction between health-related behaviors specific to viral threat (COVID-related) and more general (general health) everyday behaviors, which may have existed before the pandemic (such as diet, exercise, and regularity of health-care acquisition). The results from the structural equation model suggest that more empathetic individuals who consumed little (or no) alcohol were more likely to practice precautionary behaviors. Moreover, impulsivity and anhedonic symptoms predicted precautionary behaviors via trait health-related behaviors. The study demonstrates that the most prominent predictors of precautionary behaviors related to COVID-19 are general health behaviors. This is in line with previous research indicating that self-care improves knowledge and precautionary behaviors regarding respiratory illness and transmission (Márquez-Serrano et al., 2012). Thus, our results provide evidence for the protective properties of general health behaviors and specifically self-care in the prevention of the spread of COVID-19.

Our results join a growing body of evidence indicating that lack of empathy is linked to decreased precautionary behaviors. Relationships between empathy and adherence to precautionary behaviors have been previously found in other countries during COVID-19 and H1N1 crises (King et al., 2016; Pfattheicher et al., 2020) and among health-care workers in non-pandemic contexts (Sassenrath et al., 2016). Previous research has suggested that lack of empathy may be due to insufficient understanding of the negative consequences of individual behavior (Jonason and Krause, 2013). Indeed, precautionary health practices increased significantly when health-care professionals are reminded of the implications for others but not for themselves (Grant and Hofmann, 2011). Empathy provides an important avenue for interventions given that an experimental manipulation during the COVID-19 crisis showed that inducing empathy promotes adherence to physical distancing (Pfattheicher et al., 2020).

Impulsivity indirectly affected precautionary behaviors by prompting increased alcohol consumption and by inhibiting healthy practices. The link between impulsivity and alcohol consumption and in turn its effect on health is well documented (Dick et al., 2010; Gray and MacKillop, 2014; Griswold et al., 2018; WHO, 2018). Likewise, impulsivity has been linked to antisocial behavior (Malesza and Ostaszewski, 2016) and poor care of others (Crysel et al., 2013). Our results contribute to this literature by providing evidence that alcohol use may also be a risk for further propagating the COVID-19 virus.

More recently, research from the Netherlands found that impulse control influenced sustained compliance with mitigation measures (Reinders Folmer et al., 2020a,b). In accordance, with previous COVID-19-related evidence, impulsivity was associated to decrease in compliance with mitigation practices (Bieleke et al., 2020; van Rooij et al., 2020). Interestingly, our results demonstrate a negative covariance between impulsivity and empathy, while previous research found that those who reported psychopathic traits, characterized by high impulsivity and low empathy, were less likely to engage in preventative behaviors (Nowak et al., 2020). In keeping with previous research, our results show that individuals reporting lower impulsivity also report engaging in activities that improve their own health and may prevent the spread of the virus.

Anhedonic depression also influenced precautionary behaviors and COVID-19-resembling symptoms in an indirect way by inhibiting healthy practices. Anhedonia has been consistently linked to poorer physical health outcomes (Denollet et al., 2008), and it is thought that deficiencies in the pleasure system may influence reduced self-care behaviors (Leventhal, 2012; Kessing et al., 2014). Furthermore, stress and social isolation, which may be exacerbated by quarantine conditions, serve as a potent trigger for increased anhedonic symptoms, which in turn may lead to reduced self-care. Indeed, research during the COVID-19 pandemic has highlighted the reciprocal relationship between psychiatric illness and higher risk of being diagnosed with COVID-19 (Taquet et al., 2021). Our research extends this literature by calling attention to the importance of mental health during a pandemic.

Previous research has demonstrated inconclusive results when considering demographic factors and precautionary behaviors (Barr et al., 2008). Demographic variables were not added to the model due to various statistical restrictions (e.g., non-linear relationships and nominal variables); however, we ran some exploratory analysis on demographic variables. We did not find significant differences in precautionary behaviors by education or income levels. Our results did find that precautionary behaviors varied by age (see Supplementary Materials). Those in the 31–41 age group self-reported the least precautionary behaviors, whereas those in the 51–60 age group reported the most. This is in keeping with findings that young adults utilize the health-care system less frequently and are involved in fewer preventative health-care practices (Fortuna et al., 2009; Harris et al., 2017). However, the relationship found here was not linear, where the youngest group did not report the least and the oldest did not report the most precautionary behaviors. We further found that these behaviors varied by gender, where significant differences were found between those identifying as male and female. Both the gender and age findings might be partly due working age men being more likely to continue leaving the home for employment. However, it has been shown that men are at greater risk for COVID-19-related death due to less responsible attitudes toward the pandemic (Bwire, 2020).

Important limitations to this study must be mentioned. Firstly, due to the prevailing conditions of recommended social distancing, obtaining a representative sample was difficult. The snowball sampling technique may be biased by over-representing the academic community with a disproportionate number of highly educated individuals and participants identifying as female. Secondly, validity may have been influenced by social desirability bias as responses were collected via self-report. Additionally, the model only investigated psychological dispositional variables as predictors of precautionary behaviors and COVID-resembling symptoms. The model lacks the role played by situational variables (i.e., peer pressure, access to information, cultural values, and practices), which should be examined in future models. Moreover, other important variables such as threat perception or perception of fear for COVID-19 have proven to be crucial to predicting adherence to precautionary behaviors (Conway et al., 2020; Parlapani et al., 2020). Moreover, recent literature have highlighted the importance of human values and sharing these values in containing the COVID-19 pandemic (Wolf et al., 2020). Future research is needed to examine these relationships more thoroughly.

Additional methodological and temporal limitations must also be considered. The model did not identify a significant association between precautionary behaviors and COVID-resembling symptoms. The timing of data collection, late March to early April 2020, represents the initial stages of pandemic response in Mexico. It is reasonable to assume that COVID-resembling symptoms would be more associated with general health behaviors than pandemic-specific precautionary behaviors given the relatively early period in official response to infectious spread. Furthermore, during the data collection period, public health messages were not as consistent as they would become later, leading to concerns about ineffective or potentially dangerous actions in response to the perceived threat of the virus. As such, we opted for a mixed methods design to identify a wider spectrum of precautionary behaviors. While this approach may create measurement and scoring concerns, it provided unique insight into the behaviors taken by individuals who may not have been easily identified with closed, Likert-type items.

Other issues of internal consistency of scales need to be pointed out. Given the importance of having a time-sensitive response and the lack of validated scales at the initial stages of the pandemic, as well as the heterogeneous nature of the target phenomenon and the need for reduced scale length, some of the scales demonstrated reduced reliability. For example, the symptoms identified in the COVID-resembling symptoms scale are heterogeneous and can be associated with several maladies. Furthermore, only reversed items were used in the empathy scale to reduce survey length. While confirmatory factor analysis showed some evidence for unidimensionality of the empathy scale (see Supplementary Materials), low internal consistency scores were probably due to the low number of items used.

Despite these various limitations, the present research adds to existing literature examining psychosocial factors associated with precautionary practices in the face of a serious threat to public health like COVID-19. The historically understudied sample of Mexican respondents likewise adds heterogeneity to a growing font of international research outside of samples from Europe and the United States. These results may be informative to other epidemic and pandemic crises particularly in the Latin American and Mexican populations. Identifying the psychosocial factors that influence precautionary behavior can better inform initiatives aimed at minimizing contagion as well as elucidate some of the underlying factors that influence individual behavior during these types of medical crises. The long-term ramifications of the COVID-19 outbreak are still being examined; these types of inquiries into how best to manage such events are critical as research continues to move forward. Future studies should examine the effects of social distancing stress on mental and physical stress as well as other underlying social and environmental variables.

## Data Availability Statement

The datasets generated for this study can be found in online repositories. The names of the repository/repositories and accession number(s) can be found below: https://osf.io/b9278/.

## Ethics Statement

The studies involving human participants were reviewed and approved by Comité de Ética en Investigación de la Universidad de Sonora. The patients/participants provided their written informed consent to participate in this study.

## Author Contributions

MF-A, NC-F, and ML contributed by writing, reviewing, and editing. VC-V, MF-A, and NC-F contributed with the conceptualization and design of the study. VC-V and NC-F ran analyses and organized databases. All authors contributed to manuscript revision and read and approved the submitted version.

## Conflict of Interest

The authors declare that the research was conducted in the absence of any commercial or financial relationships that could be construed as a potential conflict of interest.
