# Supplementary material for: Psychological Predictors of Precautionary Behaviors in Response to COVID-19: A Structural Model
Source: Front Psychol. 2021 Apr 28;12:559289. doi: 10.3389/fpsyg.2021.559289 (PMC8113395; doi:10.3389/fpsyg.2021.559289)
Supplement: Supplementary file 1 [file Data_Sheet_1.pdf]

Supplemental Material

Differences in precautionary behavior by sociodemographic variables

No significant differences in precautionary behaviors were found between education or monthly income groups ($H(5) = 5.08$, $p > 0.05$; $H(5) = 9.60$, > 0.05). However, A Kruskal-Wallis one-way non parametric test revealed that there was a significant difference between gender groups ($H(3) = 39.65$, $p < 0.001$). An all-pairwise post-hoc test revealed that there were differences only between those who identified as feminine and masculine, where those who identified as non-binary reported the most precautionary behaviors (Median = 3.16), followed by those who identified as feminine (Median = 3.16), masculine orientation (Median = 2.83) and finally those who declined to identify their gender (Median = 2.79).

A non-linear trend across age ranges in relation precautionary behaviors was observed. A Kruskal-Wallis one-way non parametric test revealed that there was a significant difference between age groups ($H(4) = 18.13$, $p = 0.001$). An all-pairwise post-hoc test revealed that there were significant differences only between those in the 31 to 40 age group and 51 to 60 group. Those between 31 and 41 years exhibited the lowest reports in precautionary practices (Median = 2.83), followed by those younger than 30 (Median = 3.00), and then those in the 41 to 50 range (Median = 3.00), consequently by participants 60 and older (Median = 3.00) and finally by those between 51 and 60 (Median = 3.16).

Confirmatory Factor Analysis

To test the unidimensionality of various scales that showed low internal consistency we ran confirmatory factor analyses. A CFA assessing the factor structure was run using the statistical software EQS v6. Two main types of fit index indicators were used to evaluate whether the data

supported the proposed hypothetical model: practical (BNNFI and RMSEA), and statistical (χ^2). According to the literature and given that Mardia multivariate normalized coefficients values were greater than 7, the robust maximum likelihood method was used to run confirmatory factor analyses. Given that we wanted to assess the factor structure of the individual items and the small number of indicators per model, the individual items were used in each of the models instead of parcels.

Supplemental Figures

Supplemental Figure 1

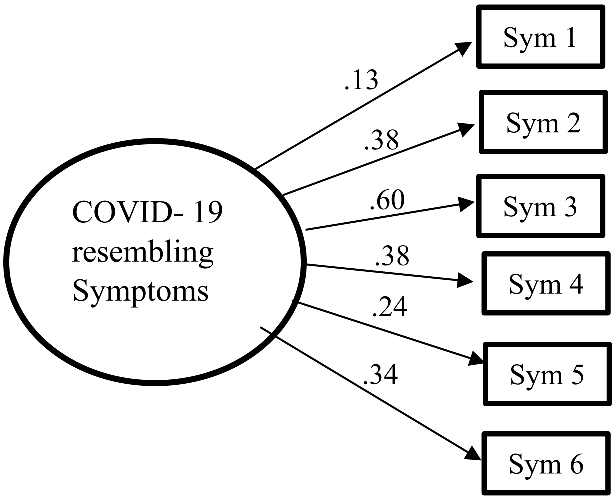

Figure 1. Confirmatory factor analysis of the COVID-19 resembling symptoms scale. Goodness of fit: $\chi2$ = 9.34 (7 *df*), *p* = .22; *BNNFI* = .98, *CFI* = .99; *RMSEA* = .02.

Supplemental Figure 2

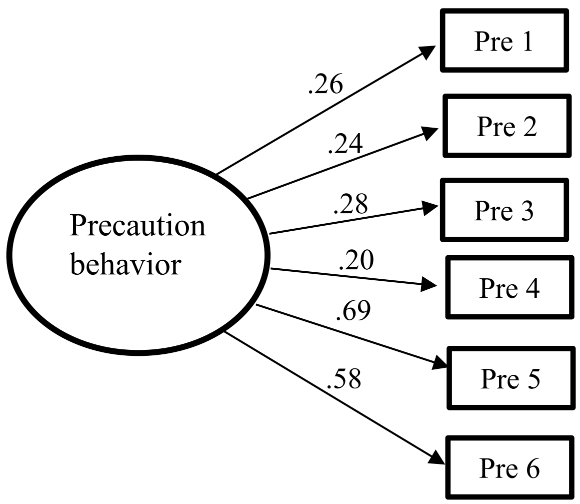

Supplemental Figure 2. Confirmatory factor analysis of the COVID-resembling symptoms scale. Goodness of fit: $\chi 2$ = 16.32 (7 *df*), *p* = .02; *BNNFI* = .90, *CFI* = .95; *RMSEA* = .05.

Supplemental Figure 3

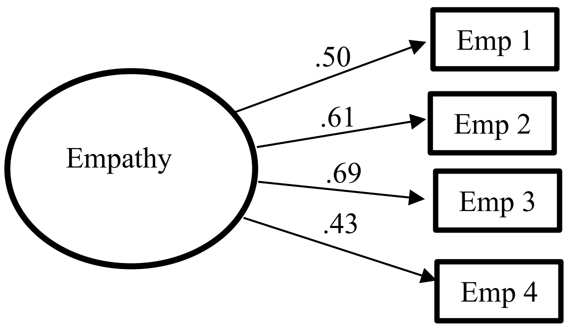

Supplemental Figure 3. Confirmatory factor analysis of the (reduced) Empathy scale. Goodness of fit: $\chi 2$ = 18.35 (2 *df*), *p* < .001; *BNNFI* = .86, *CFI* = .95; *RMSEA* = .10.

Supplemental Tables

Supplemental Table 1
Percentage of respondents reporting some degree of respiratory-disease symptoms

Symptoms	*Percentage*	*Skewness*	*Kurtosis*
Fever (38 Celsius degrees or more)	01.5	11.05	134.43
Headache	43.1	1.25	0.90
Dry cough	17.6	2.58	6.67
Loss of sense of smell	06.4	4.59	22.69
Loss of sense of taste	04.4	6.02	41.62
Stomachache	26.6	2.06	3.88
Diarrhea	12.7	3.23	10.45

Supplemental Table 2
Times respondents went out home the last three days

Times	Frequency	Percentage
Never	193	26.3
Once	264	36.0
2-3 times	167	22.8
3-5 times	41	5.6
More than 5 times	43	5.9

Table 3
Reasons for leaving home

Reasons	Frequency	Percentage
Buy food	384	52.3
Acquire medicine	75	10.2
Visit relatives	90	12.3
Doctor appointment	39	05.3
Visit friends	29	04.0
Buy alcoholic beverages	15	02.0
Exercising	54	07.4
Work	132	18.0
Other reasons	43	05.9
